# Surface-Enhanced Raman Scattering (SERS) With Silver Nano Substrates Synthesized by Microwave for Rapid Detection of Foodborne Pathogens

**DOI:** 10.3389/fmicb.2018.02857

**Published:** 2018-12-13

**Authors:** Caijiao Wei, Mei Li, Xihong Zhao

**Affiliations:** Research Center for Environmental Ecology and Engineering, Key Laboratory for Green Chemical Process of Ministry of Education, Key Laboratory for Hubei Novel Reactor & Green Chemical Technology, School of Environmental Ecology and Biological Engineering, Wuhan Institute of Technology, Wuhan, China

**Keywords:** Surface-enhanced Raman scattering, silver nanoparticles, foodborne pathogens, bioanalysis, food safety, rapid detection

## Abstract

Rapid and sensitive methods have been developed to detect foodborne pathogens, a development that is important for food safety. The aim of this study is to explore Surface-enhanced Raman scattering (SERS) with silver nano substrates to detect and identify the following three foodborne pathogens: *Escherichia coli* O157: H7, *Staphylococcus aureus* and *Salmonella*. All the cells were resuspended with 10 mL silver colloidal nanoparticles, making a concentration of 10^7^ CFU/mL, and were then exposed to 785 nm laser excitation. In this study, the results showed that all the bacteria can be sensitively and reproducibly detected directly by SERS. The distinctive differences can be observed in the SERS spectral data of the three food-borne pathogens, and the silver colloidal nanoparticles can be used as highly sensitive SERS-active substrates. In addition, the assay time required only a few minutes, which indicated that SERS coupled with the silver colloidal nanoparticles is a promising method for the detection and characterization of food-borne pathogens. At the same time, principle component analysis (PCA) and hierarchical cluster analysis (HCA) made the different bacterial strains clearly differentiated based on the barcode spectral data reduction. Therefore, the SERS methods hold great promise for the detection and identification of food-borne pathogens and even for applications in food safety.

## Introduction

For food safety management and monitoring, foodborne pathogens have always been an issue of concern as they can cause severe illness in humans via contaminated water or food (Zhao et al., 2017a). According to the data from the World Health Organization, there are many outbreaks and numerous deaths each year caused by *Escherichia coli* O157: H7, *Staphylococcus aureus*, *Salmonella*, *Listeria monocytogenes*, *Campylobacter jejuni*, and so on (Liu et al., 2017). Rapid identification and detection of pathogens are major issues for public health and food safety (Zhao et al., 2014, 2016). To date, the detection of foodborne pathogens mainly includes traditional methods, immunological methods, and molecular biology methods, but they are labor intensive, time consuming and inconvenient for onsite detection (Erik and Anna, 2007; Roda et al., 2012; Zhong and Zhao, 2017, 2018; Wei et al., 2018). It is thus necessary to explore efficient, sensitive, fast, inexpensive and accurate methods to detect and identify pathogens (Zhao et al., 2017b).

Raman spectroscopy is a scattering spectrum discovered by the Indian physicist C.V. Raman (Krishnan, 1928). The spectrum mainly provides fingerprint information of the molecular structure according to vibrational and rotational information of matter. Due to the low intensity of conventional Raman scattering (RS), it has been severely limited in many applications. Meanwhile, Surface-enhanced Raman scattering (SERS) spectroscopy avoids the problems associated with conventional RS. When the target analyte approaches or adsorbs certain rough metal (gold, silver, etc.) nanoparticle surfaces, the signals can be enhanced by many orders of magnitude compared to normal RS (Sivanesan et al., 2014). In addition, this method handles samples easily and provides the basis for non-destructive and ultra-sensitive detection of samples (Wang and Irudayaraj, 2012). SERS, as an ultrasensitive vibrational spectroscopic technique, can detect molecules on or near the surface of plasmonic nanostructures and greatly extends the role of standard RS (Wang et al., 2012). Beyond that, SERS also inherits rich chemical fingerprint information from RS, which can be conveniently made under ambient and aqueous conditions. In particular, it also gains sensitivity by plasmon-enhanced excitation and scattering and has a narrow width that is suitable for multiplex analysis (Zong et al., 2018). Therefore, SERS is rapidly emerging as a sensitive analytical tool and has been applied to many analyses and fields such as chemistry, biochemistry, microbiology, environmental sciences and so on. For example, Betz et al. (2012) detected whether melamine could be found in infant formula without the need for purification or additional equipment by using SERS. Kim et al. (2015) adopted silver nanoparticles as SERS substrates to detect C-reactive protein without using any labels; the minimum detection amounts in the buffer and in 1% serum were 0.01 and 0.1 ng mL^-1^, respectively. Sivanesan et al. (2014) developed nanostructured silver-gold bimetallic SERS substrates for selective identification of bacteria in human blood. Xu et al. (2015) applied label-free SERS to detect DNA with single-base sensitivity. SERS has the ability to identify single molecules by using their intrinsic vibrational fingerprint and can provide highly specific biochemical information about the components of bacterial cells, including proteins and peptides, polysaccharides, nucleic acids, phospholipids, etc. (Hou et al., 2014; Zheng et al., 2016; Zhao et al., 2018). Because each type of bacteria has its own specific biochemical information and can exhibit its characteristic peaks, this technique can rapidly identify good versus bad bacteria in the field based on its unique Raman fingerprint. Meisel et al. (2014) detected 19 species of the most important harmful bacteria via Raman microspectroscopy and built up a three-level classification model based on the whole amount of Raman data. After the first classifier differentiation of Gram-positive and Gram-negative bacteria by Raman spectra, two decision knots of the bacterial genus and species followed. The study showed that the accuracy of the identification results of each different step was in the range of 90.6–99.5%.

In the SERS study, it is important to bring target analyte or target molecular structure in contact with or in close proximity of the surface of metallic nanostructures (Dong et al., 2014; Huang et al., 2015; Li et al., 2017). For microorganisms and living cells, the colloidal nanoparticles are generally preferred as substrates (Culha et al., 2010). Gold and silver are two commonly used materials for the preparation of nano-metal substrates for SERS measurement. Compared with silver, gold is more expensive but produces weaker SERS enhancement than silver (Fan et al., 2011; Chuang et al., 2014). In addition, nanosilver has the following advantages: a high molar extinction coefficient, excellent optical properties and nanosilver aggregates having strong SERS effects (Wang et al., 2016). Therefore, the silver colloidal nanoparticles (AgNPs) were employed widely for bacteria detection. There are various methods for synthesizing AgNPs, such as reduction reaction methods, ultrasonic assisted reduction methods, electrolytic methods, light induction methods, thermal decomposition methods, microwave methods and so on (Huang et al., 2014). Among these, the microwave methods have the advantages of uniform reaction, easy nucleation and less pollution, and convenient and rapid synthesis; meanwhile, the prepared nanomaterials have high purity and uniform distribution (Du et al., 2015). Thus rapid microwave is an important technique for synthesizing metallic nanostructures. However, simple and green microwave methods for synthesizing highly SERS-active AgNPs have rarely been reported. Herein, the objective of this study is to evaluate the feasibility of adopting the microwave method to synthesize silver colloidal solutions as the SERS-active substrate for the detection and identification of foodborne pathogens.

## Materials and Methods

### Preparation of the Silver Colloidal Nanoparticles

In this study, the microwave heating method was used to synthesize AgNPs due to the advantages of simple operation, uniform heating, fast heating and fast preparation that it holds over other methods. In brief, 1 × 10^-3^ M of AgNO_3_ was dissolved in 200 mL double distilled water. An aliquot of 1% sodium citrate solution (10 mL) was added dropwise with continuous stirring to an aqueous silver nitrate solution. The solution was put into a microwave oven at a power of 700 W until a yellow color solution was obtained. The prepared AgNps were characterized by UV-Vis spectrophotometry (UV-1800, Shimadzu Enterprise Management, Ltd., China) under a wavelength range of 300–800 nm. At the same time, the particle size distribution of AgNps was determined by a ZEN3690 Malvern laser particle size analyzer (Malvern Instruments Ltd., United Kingdom). In order to more fully analyze the size, morphology and stability of nanoparticles, the as-synthesized AgNps was characterized by a transmission electron microscope (TEM, model JEOL JEM 1200EX) operated at 200 kV and the zeta potential of AgNps was measured by dynamic light scattering with Zetasizer Nano ZS (Malvern Instruments Ltd., United Kingdom). For TEM, samples were prepared by placing approximately 10 μL of the as-synthesized Ag colloid dispersing in water onto a TEM grid and then drying under an IR lamp.

### Preparation of Bacterial Samples

All the strains from the American Type Culture Collection (ATCC, United States) were preserved at -80°C in our laboratory until use, namely *Escherichia coli* O157:H7 (ATCC 43895), *S. aureus* (ATCC 27664) and *Salmonella* (ATCC 13076). Bacterial strains were stored at -80°C in Bacto^TM^ Tryptic Soy Broth (TSB; Becton, Dickinson and Co., Sparks, MD, United States) containing 20% glycerol, were inoculated in LB agar plates and incubated at 37°C overnight. Afterward, the pure culture was transferred to Bacto^TM^ TSB at 37°C overnight with shaking (110 rpm). The bacterial concentration was about 10^8^ CFU/mL. One milliliter of cell suspension was centrifuged at 6,000 ×*g* for 5 min and the supernatant was discarded. The cell pellets were washed three times with double distilled water, centrifuged at 6,000 ×*g* for 5 min and finally resuspended with 10 mL of the silver colloidal nanoparticles or double distilled water. Next, the solution was mixed with a vortex to obtain a mixture that was as homogeneous as possible and then stood for 3–5 min. This procedure was designed to make AgNps and bacteria adsorb each other. Transmission electron microscopy (TEM) measurements were also performed to investigate the distribution of the AgNps coated on the *Escherichia coli* O157: H7.

### SERS Measurement

**FIGURE 1:**
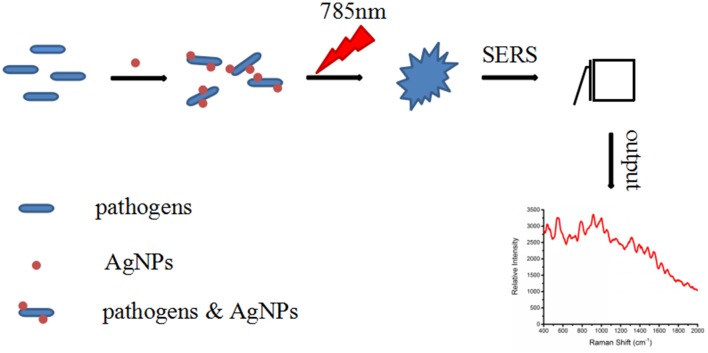
Schematic diagram of the SERS detection of bacteria with the silver colloidal nanoparticles.

In order to explore whether the AgNps that were prepared by the microwave heating method as the SERS substrate for detecting foodborne pathogens have an enhanced signal or not, the experiment was carried out as follows. All the *Escherichia coli* O157: H7, *S. aureus* and *Salmonella* samples were transferred to glass capillary tubes and then were detected using a Portable Raman Spectrometer (I0785MM0350MF, Ocean Optics Company, United States). The spectral coverage was from 400 - 2000 cm^-1^ with a 785 nm excitation light. The laser power was 70 mW and the average scan time was 4 s. Bacteria samples without AgNps were also scanned. All the procedures were carried out in three replicates. The schematic diagram for the SERS detection of foodborne pathogens with AgNPs is shown in Figure 1.

### Data Analysis

The normal Raman and SERS spectra data were analyzed by Origin software version 9.0 (Origin Lab Corporation, Northampton, MA, United States) and SPSS multivariate data analysis software (version 11.5.0, SPSS Inc., Chicago, IL, United States). The baseline correction helped to determine any difference between spectra quickly (Sundaram et al., 2013), so the pre-processing algorithms were conducted to analyze the data, for instance smoothing, normalization and second-derivative transformation. For purposes of bacterial identification, principal component analysis (PCA) and hierarchical cluster analysis (HCA) were applied in this study. Prior to the two multivariate analyses, all the spectra were smoothed in order to eliminate any high-frequency instrument background noise by averaging near data points and were then normalized to a range of 0 to 1; meanwhile, they were also subjected to second-derivative transformation to separate overlapping bands and remove baseline offsets.

## Results and Analysis

### Characterization of the AgNps

**FIGURE 2:**
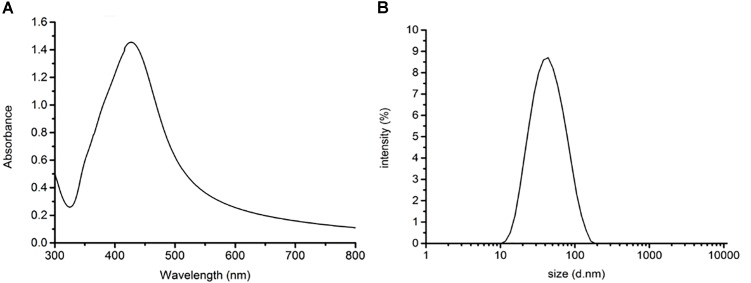
**(A)** UV-Vis absorption spectrum of AgNPs and **(B)** diameter distribution of AgNPs.

**FIGURE 3:**
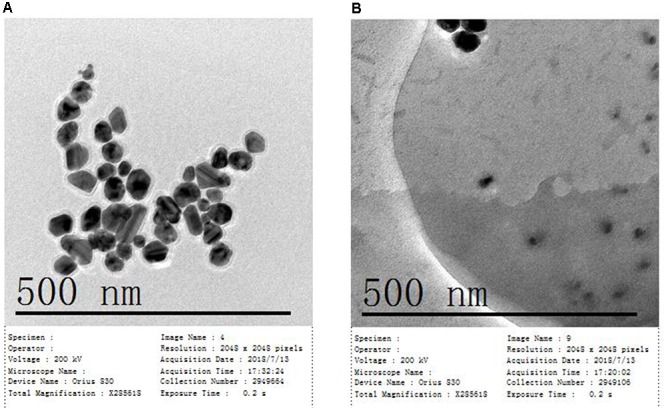
**(A)** TEM image of synthetic AgNPs and **(B)** TEM image of *Escherichia coli* O157: H7 coated with AgNPs.

The size and dispersion of AgNps have a certain effect on SERS detection, so absorption peak and particle size distribution are characterized by UV-Vis spectrophotometry and Malvern laser particle size analyzer, respectively. The characterization of the AgNPs, rapidly synthesized by green microwave technology, can be seen in Figures 2A,B. The UV-Vis spectrum of the AgNps reveals a maximum absorbance of 427 nm as is shown in Figure 2A. TEM and the corresponding size distribution revealed that the green microwave technology-synthesized AgNps have an average diameter of 65 ± 2 nm (Figures 3A, 2B). In the Figures, there are no distinct peaks in the UV-Vis and DLS spectra and no aggregation in TEM images. Thus, it can also be shown that the distribution of AgNps in the solution is relatively uniform and without aggregation. In order to verify the stability of the prepared AgNps, the zeta potential of the as-synthesized Ag colloid dispersing in water was measured three times by dynamic light scattering with Zetasizer Nano ZS. The results were -40.0, -39.3, and -40.5 mV respectively, which indicated that the as-synthesized Ag colloid dispersal in water is evenly distributed and has certain stability. According to relevant literature, the bactericidal properties of the nanoparticles are size-dependent, since the only nanoparticles that present a direct interaction with the bacteria preferentially have a diameter of approximately 1–10 nm (Morones et al., 2005; Shrivastava et al., 2007) However, in our study, the sizes of silver nanoparticles are mostly concentrated at 50–70 nm. Moreover, it takes only a few minutes from the mixing process of nano silver and bacteria to the whole process of detection. Thus, it can be stated that the proteins, purine and cell membrane of bacteria are not affected by AgNPs. Meanwhile, after placing the silver colloidal nanoparticles for 4 h, the solution was still a clear and transparent light yellow with no deposition phenomenon, which also indicates that the silver colloid has good stability. However, in bacterial SERS experiments, whether the SERS effect can generate that depends on whether the bacteria can combine effectively with AgNps. Thus, to understand the formation of the AgNP-bacteria complex, the *Escherichia coli* O157: H7 coated with silver nanoparticles was performed by TEM. It can be clearly seen from Figure 3B that the AgNPs were successfully and uniformly synthesized around the bacterial cell wall.

### SERS Spectra of Foodborne Pathogens and Structural Analysis

**FIGURE 4:**
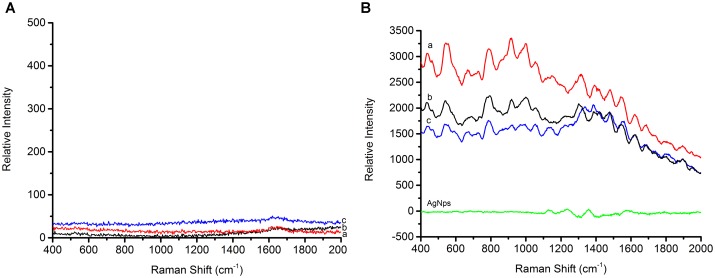
**(A)** Raman spectra of three foodborne pathogens, **(B)** SERS spectra of three foodborne pathogens (a) is *Staphylococcus aureus*, (b) is *Escherichia coli* O157: H7, and (c) is *Salmonella*, AgNPs is silver nanoparticles solution.

In order to determine how the AgNps enhance the SERS signal, equal concentrations of bacteria, both with and without AgNps, were tested and SERS spectra were recorded. As shown in Figure 4A, the foodborne pathogens without AgNps did not exhibit obvious Raman signal. However, when *Escherichia coli* O157: H7, *Staphylococcus aureus*, and *Salmonella* were coupled with AgNps, respectively, they exhibited a unique and significant Raman spectral signal (Figure 4B). These spectra were the average spectra obtained from the replicated samples. It is obvious that the SERS effect measured with the bacteria in AgNps is greatly enhanced and has stronger signal intensity than that without AgNps. It also demonstrates that SERS is capable of detecting these three foodborne pathogens with the aid of the silver colloidal nanoparticles. A comparison among the SERS spectra of *Escherichia coli* O157: H7, *S. aureus* and *Salmonella* is shown in Figures 4a–c, respectively. It can be seen from the figure that the bands of Raman vibration characteristic peaks of these three pathogens are mainly between 400 and 1650 cm^-1^. In order to further understand and distinguish these three different foodborne pathogens, all the characteristic peaks of the SERS signals are collected in \Table 1. *S. aureus* has significant Raman vibrational peaks at 437, 545, 668, 727, 788, 915, 996, 1054, 1151, 1313, 1390, 1480, 1546, and 1621 cm^-1^. The *Escherichia coli* O157: H7 SERS spectra exhibit characteristic peaks at 437, 542, 689, 792, 918, 999, 1054, 1108, 1161, 1228, 1304, 1390, 1422, 1474, 1543, and 1621 cm^-1^. Typical peaks at 437, 545, 668, 727, 788, 915, 996, 1054, 1151, 1313, 1390, 1480, 1546, and 1621 cm^-1^ are observed in *Salmonella*. In the detection of microorganisms, Raman can provide phenotypic information on signatures from cell tissues, which are mainly attributed to proteins, lipopolysaccharides, carbohydrates, nucleic acids (DNA and RNA), peptidoglycan, quinones, cytochromes, phospholipids and some endogenous biomolecules (Nelson et al., 1992). It demonstrates the vibrational information from cell structural components. Therefore, specific information can be obtained based on the structures, conformations and attributions represented by these spectra, so that the bacteria can be classified. Peaks at about 550 cm^-1^ are assigned to S-S stretching in proteins. Peaks at about 670 cm^-1^ are for the cysteine stretch model present in the protein. Peaks near 690 cm^-1^ belong to the guanine ring region of DNA/RNA. Peaks at about 730 cm^-1^ belong to the NAG component in the peptidoglycan structure of the bacterial cell wall (Jarvis and Goodacre, 2004). Peaks around at 918 cm^-1^ are attributed to the vibration of the nucleic acid and the peaks between 1220 and 1640 cm^-1^ are mainly attributed to amide I, amide II, amide III vibration and carboxylic acid stretching (Sundaram et al., 2013). The band at ∼1000 cm^-1^ is assigned to phenylalanine according to the literature (Nelson et al., 1992). The SERS peak at 918 cm^-1^ is due to C–COO- stretch (carbohydrates). A strong Raman band due to the COO-stretching vibration of proteins is observed at 1390 cm^-1^. The specific Raman peak assignments are shown in \Table 1. Comparing the Raman peaks of *Escherichia coli* O157: H7, *S. aureus* and *Salmonella*, it can be seen that there are both similarities and differences between them. For example, the number of major spectral bands of *Escherichia coli* O157: H7, *S. aureus* and *Salmonella* exhibit clear similarities, such as bands at 1054 and 1621 cm^-1^, although their relative intensities are different. However, there are also obvious differences. For instance, bands at 542 and 918 cm^-1^ are significant to *Escherichia coli* O157: H7 and *Salmonella* but not in *S. aureus*, while a band at 668 cm^-1^ is unique for *S. aureus* and *Salmonella*. However, peaks at 689 and 1422 cm^-1^ are only present in *Escherichia coli* O157: H7. Peaks at 885, 1256, and 1337 cm^-1^ are only present in *Salmonella.* To distinguish the three foodborne pathogens, the ratio of intensities of the peaks and the unique peaks can be used. Therefore, the unique and distinct vibrational spectral information of SERS can be used to identify and discriminate between different foodborne pathogens.

**Table 1:** Raman shift and tentative assignment of peaks from the SERS spectra of three foodborne pathogens.

*Staphylococcus aureus*	*Escherichia coli* O157: H7	*Salmonella*	Assignment	Reference
545	542	542	S–S stretch (proteins)	Chu et al., 2008; Fan et al., 2011
668		668	C-S stretch model of cysteine amino acid, Tyrosine (skeletal) (proteins)	Jarvis et al., 2004
	689		Guanine ring region of DNA/RNA	Sundaram et al., 2013
727		727	N acetyl D glucosamine	Jarvis and Goodacre, 2004; Sundaram et al., 2013
788	792	788	Cytosine, uracil (ring, stretch) (nucleic acids)	Schuster et al., 2000
		885	CN stretching, CON symmetric stretching; CCH rocking aliphatic	Xie et al., 2013
915	918	918	C–COO- stretch (carbohydrates)	Fan et al., 2011
996	999	990	Phenylalanine (the symmetric ring breathing mode) (proteins)	Xie et al., 2013
1054	1054	1054	CH2 stretching	Podstawka et al., 2005
	1107		CC skeletal and COC stretch from glycosidic link (carbohydrates)	Fan et al., 2011
		1133	C–N and C–C stretching (protein)	Jarvis and Goodacre, 2004; Li et al., 2015
1151	1161		(CC) stretching	Jarvis et al., 2004
	1228	1225	Amide III, lipids	Xie et al., 2013
		1256	Asymmetric PO_2-_ stretching	Xie et al., 2013
1313	1304		CH deformation	Ping et al., 2008
		1337	Nucleic acids (A, G) protein, CH deformation, C–NH2 stretching of amide II	Maquelin et al., 2000
1390	1390	1387	COO-stretching (proteins)	Schuster et al., 2000; Xie et al., 2013
	1422		Adenine, guanine CH2 deformation (lipid)	Luo and Min, 2008
1480	1474	1483	CH_2_ scissoring	Yang et al., 2011
1546	1543	1554	C = C, Asymmetric (NO_2_) stretching	Schuster et al., 2000
1621	1621	1621	Amide I (unsaturated lipids)	Schuster et al., 2000


### Reproducibility of SERS Spectra

**FIGURE 5:**
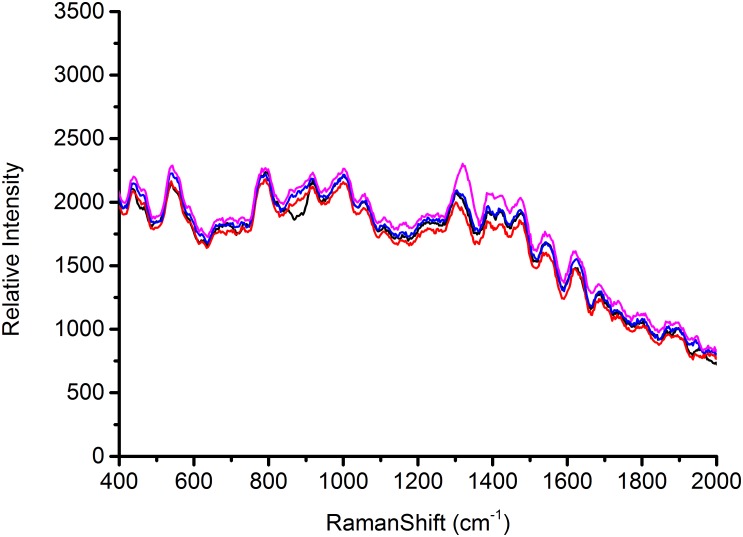
SERS spectra of *Escherichia coli* O157: H7 with four batches of prepared AgNPs.

**FIGURE 6:**
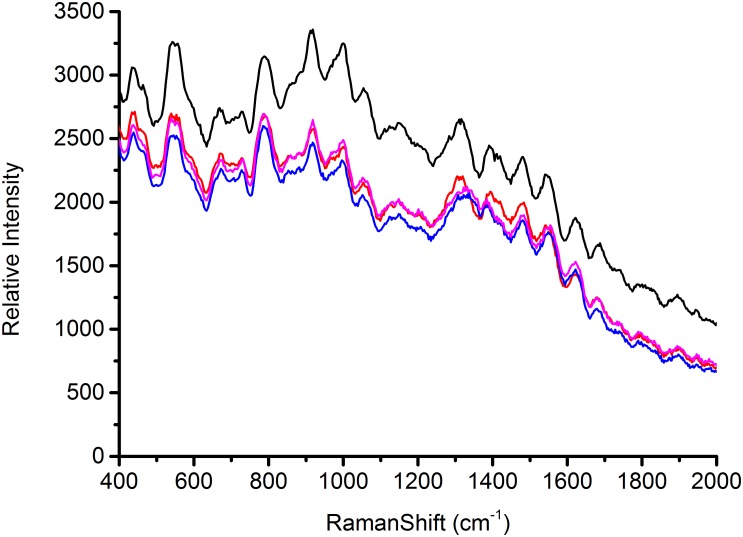
SERS spectra of *Staphylococcus aureus* with four batches of prepared AgNPs.

**FIGURE 7:**
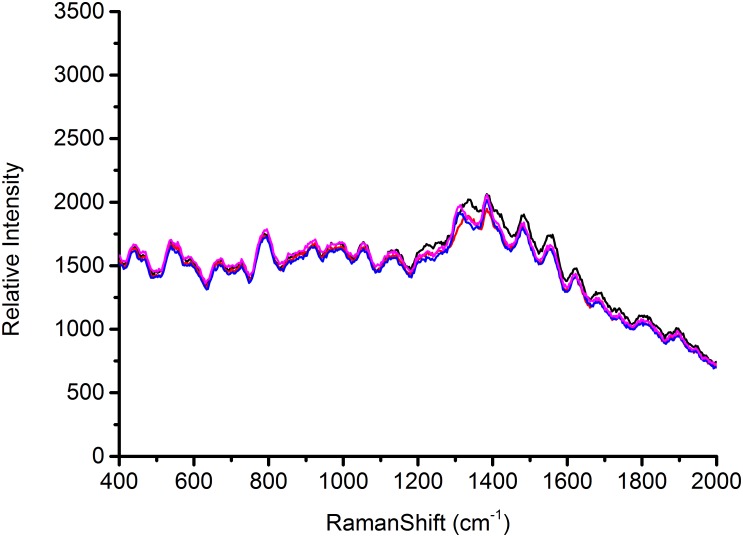
SERS spectra of *Salmonella* with four batches of prepared AgNPs.

According to the characterization of AgNps, substrates play a major role in the signal enhancement for SERS (Chu et al., 2008). Thus the reproducible SERS study using four different batches of silver nanoparticles was conducted to test *Escherichia coli* O157: H7, *S. aureus* and *Salmonella*, respectively, in this study. Each AgNps substrate was prepared independently. The three foodborne pathogens of SERS spectral reproducibility with substrates AgNps are illustrated in Figures 5–7, respectively. When bacterial samples were treated with different SERS substrates individually, the variability could be found to be common. However, in this study, the manufactured AgNps substrates did not show any change or noise in the spectra. From the figures, these three foodborne pathogens that combined four different batches of AgNps SERS spectra have a high degree of reproducibility. The results provide strong support for rapid SERS detection of foodborne pathogens.

### PCA and HCA Classification

**FIGURE 8:**
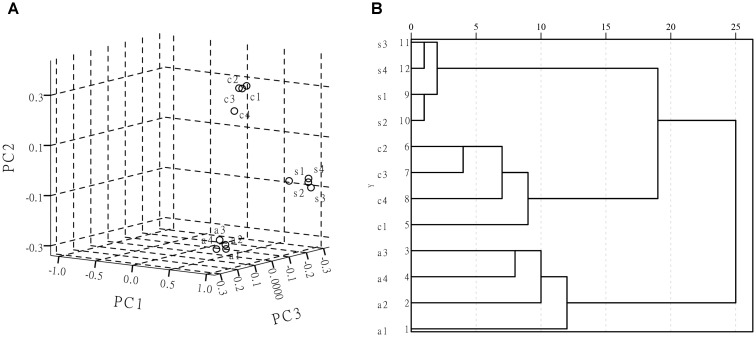
**(A)** PCA scores plot of *Escherichia coli* O157: H7, *Staphylococcus aureus*, *Salmonella*. **(B)** Composite dendrogram generated by hierarchy cluster analysis (HCA) from those three pathogenic bacteria; (s) is *Staphylococcus aureus*, (c) is *Escherichia coli* O157: H7, (a) is *Salmonella*.

Principal component analysis can greatly reduce the dimensionality of complex multivariate data to several principal components (PCs), eliminate random variation (noise) and can objectively capture the minimal spectral differences between the similar spectra (Zhang et al., 2012; Li et al., 2015). The SERS spectra are used as the inputs for the PCA model. Then the PCA projects data into the transformation space to maximize data variability, which makes it easier to observe the similarities and differences in spectra. Therefore, PCA is widely used to analyze SERS spectral data variances and develop classification models to distinguish pathogens based on the SERS spectra. Hierarchical clustering analysis is performed using measurements of distances as the standard for classification. It utilizes multiple statistical values to decide the degree of affinity between different samples. A HCA dendrogram is constructed using the Ward-linkage algorithm and squared distances, which were used to evaluate the member dissimilarity (He et al., 2008). It also utilizes the corresponding dendrograms’ multiple statistical results to discriminate and categorize the samples. At the same time, PCA results can be further corroborated. Considering that it was difficult to classify and identify microorganisms based on similar vibrational spectra, the PCA methods and HCA methods were employed to analyze and differentiate their SERS spectra acquired from bacterial samples in this study. Figure 8A shows a three-dimensional (3D) PCA plot of the first three PCs for the three bacterial strains. The PCA performed on the second derivative transformed SERS spectral data from the three foodborne pathogen samples (about 1 × 10^7^–10^8^ CFU/ml) over a range of 500–800 cm^-1^. The resulting HCA dendrogram presents a clear characterization at strain level of each analyzed foodborne pathogen. In Figure 8B, the three foodborne pathogen samples of *Escherichia coli* O157: H7, *S. aureus* and *Salmonella* can be effectively distinguished in the HCA dendrogram. The classification of HCA helps to determine the similarities and differences between groups. It can be seen from Figure 8B that the SERS spectra can mainly be categorized into three clusters and the result is consistent with that shown in PCA.

## Discussion

The AgNps were prepared by a quick and easy microwave heating method, which was used as SERS substrates to detect and discriminate *Escherichia coli* O157: H7, *S. aureus* and *Salmonella* in this study. At this point, the molecular level interpretation about SERS’ vibrational features has not been universally established, but we can make some general statements and identify these spectra based on the spectral position and varying intensities. In the given spectral region, all the foodborne pathogens have similar peaks, with some differences in frequency for certain peaks. Those results are consistent with previous reports (Zeiri et al., 2004; Sundaram et al., 2013). To allow for more precise distinction between the three foodborne pathogens SERS spectra, all the characteristic peaks in SERS spectra that can be attributed to different functional groups are collected in \Table 1. Comparing the SERS spectra of these three kinds of bacteria, we found that the three Raman are similar to unique Raman peaks in themselves. *S. aureus* and *Salmonella* both have Raman peaks at 727 cm^-1^, which are attributed to N-acetylglucosamine (NAG) in peptidoglycans. This result also confirms that the NAG exists in both Gram-negative and Gram-positive bacteria, whereas for *Escherichia coli* O157: H7 this region is represented by a broad and weak peak. The similar results were obtained in the study of pathogen identification using a portable Raman spectroscopy system (Luo and Min, 2008). Moreover, all of them have many differences, which mainly come from the quantity and distribution of cellular components such as proteins, phospholipids, nucleic acids, and carbohydrates. For instance, *Escherichia coli* O157: H7 has unique Raman peaks at 689, 1107, 1161, 1304, and 1422 cm^-1^, *S. aureus* has its own unique Raman peaks at 966, 1151, 1313, and 1480 cm^-1^, while *Salmonella* has their own unique Raman peaks at 885, 1133, 1256, and 1337 cm^-1^. Meanwhile, there is also an obvious difference in the intensity of SERS vibrational peaks among these three foodborne pathogens. However, the spectral information of these three pathogens is not completely coincident with other studies. Comparing with other studies (Xie et al., 2013; Wang et al., 2016), they appear to be different peaks. It indicates that these spectra only presented ingredient information activated by metallic nanosilver.

In this study, a rapid SERS technique coupled with silver colloidal nanoparticles as substrates has been explored to identify the foodborne pathogens. Compared to traditional detection methods, this method is faster and easier to perform and has a high degree of reproducibility. All the foodborne pathogens have similar peaks, with some difference in frequency for certain peaks in the given spectral region. The spectra showed largely similar peaks, such as those at 542, 918, 1054, and 1621 cm^-1^, but they also have their unique peaks that can easily be distinguished. In addition, it is difficult to classify and identify microorganisms due to similar vibrational spectra, so multivariate statistical analysis of PCA and HCA were applied to explore the data and identify individual groups based on differences in the SERS spectra in this study.

## Conclusion

The SERS has great prospects for application in detection of foodborne pathogens. If this method is further applied, quantitative detection of viable cells from dead cells, identifying different foodborne pathogen species and subspecies level, and then establishing a bacterial SERS database could be next steps. We believe that SERS will be more universally applicable in the area of food microbiology and also provides a sensitive and efficient tool for food safety control. So far, it is still very challenging to apply SERS techniques to detect pathogens for qualitative and quantitative analyses in complex media (food). If SERS can be integrated with chemometrics methods and spectral data analysis as well as with the development of micro-Raman spectrometers and nanosubstrates, and determine the best conditions to obtain reproducible results in complex food matrices or separate the bacteria from the complex food substrate via some filter membranes, there could be further advances not only in food, but also in the human health sectors.

## Author Contributions

XZ designed this study and wrote the manuscript. CW and ML finished the experiments and collected the data. All authors read and approved the final manuscript.

## Conflict of Interest Statement

The authors declare that the research was conducted in the absence of any commercial or financial relationships that could be construed as a potential conflict of interest.
